# Extra upper limb practice after stroke: a feasibility study

**DOI:** 10.1186/s40814-019-0531-5

**Published:** 2019-12-30

**Authors:** Emma J. Schneider, Louise Ada, Natasha A. Lannin

**Affiliations:** 1 School of Allied Health (Occupational Therapy), College of Science, Health and Engineering, La Trobe University, Plenty Road and Kingsbury Drive, Melbourne, Victoria 3086 Australia; 2 Occupational Therapy Department, Alfred Health, 55 Commercial Road, Melbourne, Victoria 3004 Australia; 3 Discipline of Physiotherapy, Faculty of Health Sciences, The University of Sydney, 75 East Street, Lidcombe, New South Wales 2141 Australia; 4 Department of Neuroscience, Central Clinical School, Monash University, 99 Commercial Road, Melbourne, Victoria 3004 Australia

**Keywords:** Rehabilitation, Occupational therapy, Physical therapy, Task-specific motor training

## Abstract

**Background:**

There is a need to provide a large amount of extra practice on top of usual rehabilitation to adults after stroke. The purpose of this study was to determine if it is feasible to add extra upper limb practice to usual inpatient rehabilitation and whether it is likely to improve upper limb activity and grip strength.

**Method:**

A prospective, single-group, pre- and post-test study was carried out. Twenty adults with upper limb activity limitations who had some movement in the upper limb completed an extra hour of upper limb practice, 6 days per week for 4 weeks. Feasibility was measured by examining recruitment, intervention (adherence, efficiency, acceptability, safety) and measurement. Clinical outcomes were upper limb activity (Box and Block Test, Nine-Hole Peg Test) and grip strength (dynamometry) measured at baseline (week 0) and end of intervention (week 4).

**Results:**

Of the 212 people who were screened, 42 (20%) were eligible and 20 (9%) were enrolled. Of the 20 participants, 12 (60%) completed the 4-week program; 7 (35%) were discharged early, and 1 (5%) withdrew. Participants attended 342 (85%) of the possible 403 sessions and practiced for 324 (95%) of the total 342 h. In terms of safety, there were no study-related adverse events. Participants increased 0.29 blocks/s (95% CI 0.19 to 0.39) on the Box and Block Test, 0.20 pegs/s (95% CI 0.10 to 0.30) on the Nine-Hole Peg Test, and 4.4 kg (95% CI 2.9 to 5.9) in grip strength, from baseline to end of intervention.

**Conclusions:**

It appears feasible for adults who are undergoing inpatient rehabilitation and have some upper limb movement after stroke to undertake an hour of extra upper limb practice. The magnitude of the clinical outcomes suggests that further investigation is warranted and this study provides useful information for the design of a phase II randomized trial.

**Trial registration:**

Australian and New Zealand Clinical Trial Registry (ACTRN12615000665538).

## Background

Upper limb activity is necessary for participation in activities of daily living [1]. More than 80% of stroke survivors have motor impairments that can include changes to muscle strength as well as difficulty in controlling movement [2]. This decrease in muscle strength and control results in a person needing assistance to complete basic daily activities [1]. Upper limb rehabilitation, therefore, aims to improve both muscle strength and movement control [3] and is structured to provide repetitive upper limb practice of specific tasks that are challenging, progressive and skill-based [4, 5]. Yet the recovery of upper limb activity after stroke is often poor [6] and stroke survivors perceive that their time spent in upper limb rehabilitation was not sufficient [7].

There is high-level evidence that an increase in the amount of supervised rehabilitation improves motor outcome for stroke survivors [4, 8–10], with four systematic reviews finding small to moderate effect sizes [8–10]. One review investigated how much extra rehabilitation was required to produce a benefit and found that a 240% increase in the amount of usual rehabilitation was needed to ensure that the extra rehabilitation improved activity [10]. For example, if 25 min of upper limb rehabilitation per day is usual, an extra 60 min (a total of 85 min per day) would need to be provided to result in an improvement in upper limb activity. This is almost three times the amount of usual rehabilitation and a large amount of extra practice.

The challenge now is to determine a feasible way to provide a large amount of extra practice taking into account staff and resource constraints. Most studies to date have delivered extra rehabilitation in one-on-one sessions outside the usual rehabilitation service [11–21]. This model of delivery, however, is not an efficient way to increase the amount of usual rehabilitation in an inpatient rehabilitation service. The potential to provide extra rehabilitation without using one-on-one supervised sessions has been explored using various strategies such as gaming, group practice or homework [22–26]. We propose to investigate using largely self-directed practice within inpatient rehabilitation as a way of increasing the amount of upper limb practice in the subacute phase after stroke. In preparation for a large, fully-powered randomized trial, it is important to understand the feasibility of recruitment, delivering the intervention and collecting the outcome measures. Therefore, the primary questions of this study were:

1. Is it feasible (in terms of recruitment, intervention and measurement) for people who are undergoing inpatient rehabilitation and have some movement in the upper limb after stroke to undertake an extra hour of upper limb practice, 6 days per week for 4 weeks?
2. Is the extra practice likely to improve upper limb activity and grip strength?

## Method

### Design

**Fig. 1:**
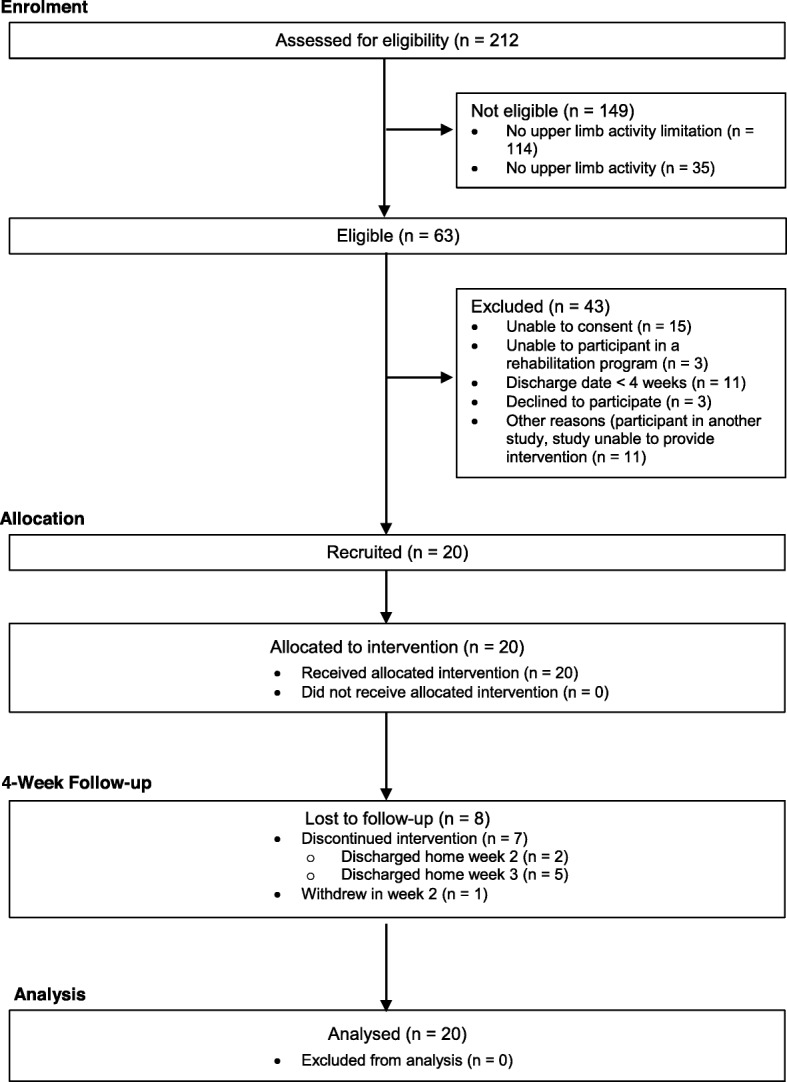
CONSORT diagram showing the design and flow of participants through each stage of the study

A prospective, single-group, pre- and post-test study was conducted at a metropolitan inpatient rehabilitation hospital in Melbourne, Australia. The participants received extra upper limb practice for 4 weeks. Outcomes were measured at baseline (week 0) and at the end of intervention (week 4). The design of the study is presented in Fig. 1. Outcome measures were collected by occupational therapists trained in the procedures who were not blinded to the aims of the study. University and hospital human research ethics committees approved this study. All participants gave written informed consent before data collection began.

### Setting

The study was conducted in one sub-acute rehabilitation hospital that has > 25 beds dedicated to multidisciplinary inpatient rehabilitation after stroke.

### Participants and therapists

Consecutive patients admitted for inpatient rehabilitation with stroke between July 2015 and June 2016 were screened for eligibility by a researcher within 72 h of admission. Patients were eligible if they had a medical diagnosis of stroke, were aged over 18 years, had an upper limb activity limitation (defined as < 54 blocks on the Box and Block Test which is a 20% reduction in the normal scores of adults aged 20–80 years) [27], and had some upper limb activity (> Grade 1 wrist extension and > Grade 3 shoulder elevation on manual muscle testing) in order to be able to carry out the practice [28]. Patients were excluded if they had severe cognitive and/or language defects (Mini Mental Status Examination score ≤ 24) [29], had any medical condition that precluded them participating in a rehabilitation program aimed at upper limb activity, or had a discharge date that precluded them completing the 4-week program. For patients who were initially ineligible (no upper limb activity), screening was repeated weekly to establish if they became eligible. Age (year), sex (number male), time since stroke (days), side of hemiplegia (number right), living situation (lives alone), education (attended university), cognition (Mini Mental Status Examination, 0–30) [29], unilateral special neglect (Albert’s Line Cancellation Test, number of lines left uncrossed) [30], loss of light touch sensation (none/some/complete), spasticity (Tardieu Scale Quality of Muscle Reaction, 0–5) [31], contracture (range of motion at the wrist and elbow), complexity of rehabilitation needs (Rehabilitation Complexity Scale-Extended, 0–20) [32], and ability to pick up a cup unaided (number) and walk unaided (number) were collected at baseline to describe the sample.

Occupational therapists overseeing the extra upper limb practice all had experience in neurological rehabilitation and were trained in task-specific motor training and the trial intervention prior to study commencement. One therapist was involved in overseeing the extra upper limb practice, with incidental support from two additional therapists.

### Intervention

Participants undertook an extra hour of upper limb practice, 6 days a week (Monday to Saturday) for 4 weeks, consisting of two self-directed programs designed to be used by adults with stroke: the Graded Repetitive Arm Supplementary Program (GRASP) and the AbleX [22, 23]. GRASP is a self-directed arm and hand program that incorporates strengthening exercises, part practice and practice of whole upper limb activities [22]. GRASP has three levels of difficulty. The level of difficulty prescribed was determined by participant performance on weekly clinical outcome measures and ability to complete half of the tasks at the maximum number of set repetitions [22]. The therapist provided the participant with one of six GRASP kits (manual and equipment) at the start of each session. AbleX is a computer-based upper limb program that was set up by the therapist on a laptop. Participants hold a controller in their affected hand or bilaterally to play a range of computer games designed to promote target-hitting [23]. The computer system provides participants with immediate feedback on their performance (accuracy), activity time (adherence) and exercise intensity [23].

Therapists provided direction and encouragement to practice, set-up the equipment, checked the quality of the practice, and progressed the difficulty of practice to ensure the level of challenge was always high. The amount of support was gradually reduced once the participant could follow the self-directed programs. To set up the equipment, the therapist provided the participant with a pre-packed GRASP kit or laptop. The extra practice could be undertaken at any time during usual rehabilitation hours (8 a.m. to 5 p.m.), individually or in a group, in the therapy area or a common space in the ward. The time of the extra practice session was scheduled on the participant’s timetable to ensure the participant was ready for each session. Participants were encouraged to complete the required amount of daily practice but could choose to practice for greater or less than 60 min per session. The amount of practice and session duration was tracked and recorded by the participant with assistance from the therapist using a stopwatch and paper diary.

No other aspects of the multidisciplinary rehabilitation were changed. The amount of usual upper limb rehabilitation that was scheduled on the participant’s timetable by the multidisciplinary rehabilitation team was collected. Usual upper limb rehabilitation could include a combination of individual and group sessions provided by occupational therapists and/or physiotherapists targeting task-specific motor training of the affected upper limb.

### Outcome measures

#### Feasibility

The feasibility of the study involved examining recruitment, intervention (adherence, efficiency, acceptability, and safety) and measurement. The feasibility of recruitment was determined by calculating the proportion of enrolled patients from the population who were screened for eligibility. Feasibility of the intervention was determined by examining adherence (the number of sessions attended as a proportion of the number of possible sessions), efficiency (the amount of practice as a proportion of total minutes), acceptability (participants yes/no responses to 5 statements about the training and rating of their acceptability from 0 to 5, Table 2), and safety (the number of adverse events such as fatigue, illness, muscle soreness, or injuries as a proportion of the number of sessions attended). If required, an interpreter or non-verbal communication assisted the participant. The feasibility of measurement involved examining how many participants could be measured for all outcomes.

#### Clinical

Clinical outcomes were upper limb activity and grip strength. Upper limb activity was measured using the Box and Block Test (number of blocks) and the Nine-Hole Peg Test (s). Grip strength (kg) was measured using dynamometry. The Box and Block Test is a timed test of the ability to grasp and release. The instructions for the test were standardized according to Mathiowetz et al. [27]. Participants were asked to pick up and move one block at a time over a barrier to the other side of the box as quickly as possible. The ability to grasp and release was transferred to a rate of performance by dividing the number of blocks moved by 60 s (number of blocks/s).

The Nine-Hole Peg Test is a timed test of the ability to grasp, manipulate and place small objects with one hand. The instructions for the test were modified to incorporate additional stopping points [33, 34]. Participants were asked to pick up the 9 pegs one at a time and place them in the holes until all nine holes were filled; then remove the 9 pegs one at a time and return them to the tray. The participants were told not to continue the test if they had placed zero pegs into the holes at 60 s [33]. The participants were told not to continue the test if they had not completed the test (placed and removed all 9 pegs) in 120 s [34]. The number of pegs moved was quantified as 0–18 pegs; either 0–9 pegs placed into the holes or 10–18 pegs returned to the tray. The score was then transferred to a rate of performance by dividing the number of pegs moved by the number of seconds to complete or stop the test (pegs/s).

Dynamometry of maximum voluntary contraction of grip measures the strength of muscles in the forearm and hand. The instructions for the test were standardized according to Horowitz [35]. Grip strength was quantified by the number of kilograms achieved. If the participant could register some strength but not enough to reach the first increment on the dynamometer (at 2 kg), the score was recorded as 1 kg.

### Sample size

Due to the nature of a feasibility study, a formal sample size calculation was not performed [36]. We aimed to recruit 20 participants as this was considered an adequate number to assess the feasibility [37].

### Data analysis

For participant characteristics and feasibility outcomes, descriptive statistics are presented as mean (SD) or number (%). For clinical outcomes, paired between-time differences (week 4 minus week 0) are presented as mean difference (95% CI). When a participant was discharged home or from the study before week 4, a measure was taken at this time.

## Results

### Characteristics of participants

Twenty participants aged 63 (SD 17) years, of which 11 (55%) were men, participated in the study. Characteristics of participants are presented in Table 1. Usual upper limb rehabilitation was scheduled for a mean of 37 (SD 26) min per day with 4 (20%) participants scheduled to receive no upper limb rehabilitation.

**Table 1:** Baseline characteristics of participants

Characteristic	(*n* = 20)
Age (year), mean (SD)	63 (17)
Sex, *n* male (%)	11 (55)
Time since stroke (day), mean (SD)	38 (87)
Side of hemiplegia, *n* right (%)	12 (60)
Living situation, *n* lives alone (%)	9 (45)
Education, *n* attended university (%)	9 (45)
Cognition (MMSE, 0–30), mean (SD)	28 (2)
Neglect (Albert’s Line Cancelation Test), *n* (%)	2 (10)
Loss of light touch sensation, *n* (%)	
None	18 (90)
Some	2 (10)
Complete	0 (0)
Spasticity (Tardieu Scale Quality of Muscle Reaction, 0–5), mean (SD)	
Wrist flexors	0.15 (0.38)
Biceps	0.2 (0.51)
Contracture upper limb, *n* (%)	3 (15)
Complexity of rehabilitation needs (RCS, 0–20), mean (SD)	12 (2)
Grasps unaided, *n* (%)	10 (50)
Walks unaided, *n* (%)	2 (10)

*MMSE* Mini-Mental Status Exam, *RCS* Rehabilitation Complexity Scale-Extended

### Feasibility

#### Recruitment

Over an 11-month period, 212 people were screened, 42 (20%) were eligible, and 20 (9%) were enrolled. In terms of retention, at week 4, 7 (35%) participants had already been discharged home and one (5%) had withdrawn (co-enrolled in another study and reported fatigue). Participants completed the extra upper limb practice program for a mean of 3 (SD 1) weeks. The flow of participants through the study is presented in Fig. 1.

#### Intervention

Removing the 77 sessions missed due to early discharge of seven participants from the study, there were a possible 403 sessions. Adherence to the intervention was 85% (i.e., 342 out of a possible 403 sessions). Forty-five (11%) sessions were missed because of non-attendance (illness, fatigue, visitors); and 15 (4%) sessions were missed because the participant withdrew. Efficiency of the intervention was 95%; i.e., participants completed 324 h of practice during a total of 342 h. Participants undertook a mean of 57 (SD 9) min of extra upper limb practice during a mean session of 73 (SD 10) min. Acceptability of the intervention is presented in Table 2. Overall, the participants were satisfied (4.8 out of 5.0) with their extra practice. In terms of safety, the incidence of fatigue, illness, or muscle soreness during the 342 intervention sessions was 40 (12%); 32 (9%) reports of fatigue; 4 (1%) reports of illness; 4 (1%) reports of localized muscle soreness in the affected arm. There were no injuries or serious adverse events (study related or otherwise).

**Table 2:** Acceptability of the extra rehabilitation

Acceptability	(*n* = 20)
Would you recommend this program to a friend who had suffered a stroke and couldn’t move their arm normally, number yes (%)	19 (95)
On average, was the program, number yes (%):	
Too much practice/exercise for your arm and hand?	1 (5)
Too little practice/exercise for your arm and hand?	1 (5)
Just enough practice/exercise for your arm and hand?	18 (90)
Did the practice make you tired, number yes (%)	8 (40)
Did the practice make you so tired that you wanted to stop, number yes (%)	3 (15)
How satisfied are you with the extra practice you received (0–5*), mean (SD)	4.8 (0.5)

*Where 0 is ‘strongly not satisfied at all’ and 5 is ‘very satisfied’

#### Measurement

Clinical outcomes were collected from all 20 (100%) participants at week 4 or prior to discharge home or withdrawal.

### Clinical

The group clinical outcomes are presented in Table 3. There was a mean 0.29 blocks/s (95% CI 0.19 to 0.39) increase on the Box and Block Test from baseline to end of intervention. There was a mean 0.20 pegs/s (95% CI 0.10 to 0.30) increase on the Nine-Hole Peg Test from baseline to end of intervention. There was a mean 4.4 kg (95% CI 2.9 to 5.9) increase in grip strength from baseline to end of intervention.

**Table 3:** Mean (SD) for clinical outcomes at each time, mean (95% CI) difference between times and reference values for healthy adults

Clinical outcome	Reference value	Times		Difference between times
		Week 0	Week 4	Week 4 minus Week 0
Box and Block Test (blocks/s)	1.3 [27]	0.29 (0.25)	0.58 (0.33)	0.29 (0.19 to 0.39)
Nine-Hole Peg Test (pegs/s)	1.0 [34]	0.18 (0.20)	0.37 (0.33)	0.20 (0.10 to 0.30)
Grip strength (kg)	32 [38]	12 (11)	17 (11)	4 (3 to 6)

## Discussion

This study demonstrates that it appears feasible for people who are undergoing inpatient rehabilitation and have some movement in the upper limb after stroke to undertake an extra hour of upper limb practice, 6 days per week until discharge or for up to 4 weeks. Participants attended the majority of sessions, practiced for the majority of session duration, rated the acceptability of the intervention as high, and reported a low number of adverse events during the extra upper limb practice. The change observed in the clinical outcomes suggests a promising improvement in upper limb activity and grip strength above what might normally be expected [39]. For example, it has been suggested that time alone accounts for 16% improvement in impairments over 6–10 weeks [39] compared with our 42% improvement in grip strength and 100% improvement in upper limb activity over 4 weeks.

This study provided evidence that extra practice was feasible; however, this was not provided within the usual resources provided within the inpatient rehabilitation unit. The participants were often unavailable during usual working hours, either completing usual daily activities (shower, dress, eat), engaged in usual rehabilitation, resting, or with family/visitors. Therefore, the extra upper limb practice was often undertaken after usual rehabilitation and before dinner (4.30–5.30 p.m.) and within the common space in the ward to reduce transportation and where nursing staff could ensure the safety of the participants during self-directed practice. Seventy-two percent of the self-directed practice was undertaken in a group in the ward. We recommend that future trials designed to deliver extra upper limb practice to adults undergoing inpatient rehabilitation consider (i) using a group format and (ii) the timing of sessions.

Adults undergoing inpatient rehabilitation were able to undertake a mean of 57 min of extra upper limb practice during a mean session of 73 min, on top of a mean of 37 min of usual upper limb rehabilitation per day. These results are comparable to the findings of the Schneider et al. [10] systematic review; 37 min of usual upper limb rehabilitation per day and an extra 73 min of extra upper limb rehabilitation per day. This equates to a 200% increase in the amount of usual rehabilitation, only slightly less than the suggested 240% increase [10]. Furthermore, reports of fatigue, illness, or muscle soreness was low (12%) and consistent with other studies in similar settings for adults after stroke [40, 41].

There are limitations to this study. First, the use of one AbleX device limited the number of adults who could complete the extra upper limb practice program at one time and in some circumstances, recruitment was stopped to ensure delivery of the intervention. While the enrollment of 48% of the eligible participants is comparable to other studies [42], access to more than one AbleX program, or use of the GRASP program alone, may improve the recruitment of future studies. Second, the high rate of early discharge; participants completed the extra upper limb practice program for a mean of 3 weeks, delivered over a mean of 20 sessions. This suggests that future trials either need to continue the program after discharge or reduce the duration from 4 to 3 weeks. Third, while the clinical outcomes suggest a promising improvement in upper limb activity and grip strength, it must be noted that all participants had some movement at the time of recruitment, which suggests they were capable of recovery due to having had an intact corticospinal tract [43]. Fourth, the use of assessors who were aware of the study aims may have led to bias estimates of clinical outcomes.

## Conclusion

It appears feasible for adults who are undergoing inpatient rehabilitation and have some upper limb movement after stroke to undertake an extra 1 h of upper limb practice, 6 days per week until discharge or for up to 4 weeks. The extra upper limb practice program was feasible when delivered outside usual therapy time and in a group in the common space of the ward. Clinical outcomes suggest a promising improvement in upper limb activity and grip strength. Further investigation is warranted and this study provides useful information for the design of a phase II randomized trial.

## Data Availability

The datasets used and/analyzed during the current study are available from the corresponding author on reasonable request.

## References

[1] Australian Institute of Health and Welfare, Australian Government Department of Health (2013). Stroke and its management in Australia: an update. Cardiovascular disease series 37.

[2] Geyh Szilvia, Cieza Alarcos, Schouten Jan, Dickson Hugh, Frommelt Peter, Omar Zaliha, Kostanjsek Nenad, Ring Haim, Stucki Gerold (2004). ICF Core Sets for stroke. Journal of Rehabilitation Medicine.

[3] Carr JH, Shepherd RB (2003). Neurological rehabilitation: optimising motor performance.

[4] Lohse KR, Lang CE, Boyd LA (2014). Is more better? Using metadata to explore dose-response relationships in stroke rehabilitation. Stroke..

[5] Stroke Foundation. Clinical guidelines for stroke management. Australia 2010. Available from: https://informme.org.au/guidelines/clinical-guidelines-for-stroke-management-2010.

[6] Dean C, Mackey F (1992). Motor assessment scale scores as a measure of rehabilitation outcome following stroke. Aust J Physiother.

[7] Barker RN, Brauer SG (2005). Upper limb recovery after stroke: the stroke survivors’ perspective. Disabil Rehabil..

[8] Veerbeek JM, van Wegen E, van Peppen R, van der Wees PJ, Hendriks E, Rietberg M (2014). What is the evidence for physical therapy poststroke? A systematic review and meta-analysis. PloS One..

[9] Veerbeek JM, Koolstra M, Ket JC, van Wegen EE, Kwakkel G (2011). Effects of augmented exercise therapy on outcome of gait and gait-related activities in the first 6 months after stroke: a meta-analysis. Stroke..

[10] Schneider EJ, Lannin NA, Ada L, Schmidt J (2016). Increasing the amount of usual rehabilitation improves activity after stroke: a systematic review. J Physiother..

[11] Burgar CG, Lum PS, Scremin AM, Garber SL, Van der Loos HF, Kenney D (2011). Robot-assisted upper-limb therapy in acute rehabilitation setting following stroke: Department of veterans affairs multisite clinical trial. J Rehabil Res Dev..

[12] Cooke EV, Tallis RC, Clark A, Pomeroy VM (2010). Efficacy of functional strength training on restoration of lower-limb motor function early after stroke: phase I randomized controlled trial. Neurorehabil Neural Repair..

[13] Donaldson C, Tallis R, Miller S, Sunderland A, Lemon R, Pomeroy V (2009). Effects of conventional physical therapy and functional strength training on upper limb motor recovery after stroke: a randomized phase II study. Neurorehabil Neural Repair..

[14] GAPS (2004). Can augmented physiotherapy input enhance recovery of mobility after stroke? A randomized controlled trial. Clin Rehabil..

[15] Han C, Wang Q, Meng PP, Qi MZ (2013). Effects of intensity of arm training on hemiplegic upper extremity motor recovery in stroke patients: a randomized controlled trial. Clin Rehabil..

[16] Kim M, Cho K, Lee W (2014). Community walking training program improves walking function and social participation in chronic stroke patients. The Tohoku journal of experimental medicine..

[17] Kwakkel G, Wagenaar RC, Twisk JW, Lankhorst GJ, Koetsier JC (1999). Intensity of leg and arm training after primary middle-cerebral-artery stroke: a randomised trial. Lancet..

[18] Lincoln NB, Parry RH, Vass CD (1999). Randomized, controlled trial to evaluate increased intensity of physiotherapy treatment of arm function after stroke. Stroke..

[19] Partridge C, Mackenzie M, Edwards S, Reid A, Jayawardena S, Guck N (2000). Is dosage of physiotherapy a critical factor in deciding patterns of recovery from stroke: a pragmatic randomized controlled trial. Physiother Res Int..

[20] Rodgers H, Mackintosh J, Price C, Wood R, McNamee P, Fearon T (2003). Does an early increased-intensity interdisciplinary upper limb therapy programme following acute stroke improve outcome?. Clin Rehabil..

[21] Ross LF, Harvey LA, Lannin NA (2009). Do people with acquired brain impairment benefit from additional therapy specifically directed at the hand? A randomized controlled trial. Clin Rehabil..

[22] Harris JE, Eng JJ, Miller WC, Dawson AS (2009). A self-administered Graded Repetitive Arm Supplementary Program (GRASP) improves arm function during inpatient stroke rehabilitation: a multi-site randomized controlled trial. Stroke..

[23] Hijmans JM, Hale LA, Satherley JA, McMillan NJ, King MJ (2011). Bilateral upper-limb rehabilitation after stroke using a movement-based game controller. J Rehabil Res Dev..

[24] Thomson K, Pollock A, Bugge C, Brady M (2014). Commercial gaming devices for stroke upper limb rehabilitation: a systematic review. Int J Stroke.

[25] English C, Bernhardt J, Crotty M, Esterman A, Segal L, Hillier S (2015). Circuit class therapy or seven-day week therapy for increasing rehabilitation intensity of therapy after stroke (CIRCIT): a randomized controlled trial. Int J Stroke.

[26] Page SJ, Levin L, Hermann V, Dunning K, Levine P (2012). Longer versus shorter daily durations of electrical stimulation during task-specific practice in moderately impaired stroke. Arch Phys Med Rehabil..

[27] Mathiowetz V, Volland G, Kashman N, Weber K (1985). Adult norms for the Box and Block Test of manual dexterity. Am J Occup Ther..

[28] Kendall FP, McCreary EK, Provance PG (1993). Muscles: Testing and Function.

[29] Folstein MF, Folstein SE, McHugh PR (1975). “Mini-mental state”. A practical method for grading the cognitive state of patients for the clinician. J Psychiatr Res..

[30] Albert ML (1973). A simple test of visual neglect. Neurology..

[31] Gracies JM, Marosszeky JE, Renton R, Sandanam J, Gandevia SC, Burke D (2000). Short-term effects of dynamic lycra splints on upper limb in hemiplegic patients. Arch Phys Med Rehabil..

[32] Turner-Stokes L, Scott H, Williams H, Siegert R (2012). The rehabilitation complexity scale--extended version: detection of patients with highly complex needs. Disabil Rehabil..

[33] Chen HM, Chen CC, Hsueh IP, Huang SL, Hsieh CL (2009). Test-retest reproducibility and smallest real difference of 5 hand function tests in patients with stroke. Neurorehabil Neural Repair..

[34] Mathiowetz V, Weber K, Kashman N, Volland G (1985). Adult norms for the Nine Hole Peg Test of finger dexterity. Am J Occup Ther.

[35] Horowitz BP, Tollin R, Cassidy G (1997). Collection of normative data with community dwelling elders. Phys Occup Ther Geriatr.

[36] Tickle-Degnen L (2013). Nuts and bolts of conducting feasibility studies. Am J Occup Ther..

[37] Billingham SA, Whitehead AL, Julious SA (2013). An audit of sample sizes for pilot and feasibility trials being undertaken in the United Kingdom registered in the United Kingdom Clinical Research Network database. BMC Med Res Methodol..

[38] Massy-Westropp NM, Gill TK, Taylor AW, Bohannon RW, Hill CL. Hand Grip Strength: age and gender stratified normative data in a population-based study. BMC Res Notes. 2011;4:127. 10.1186/1756-0500-4-127 PMC3101655 21492469

[39] Kwakkel Gert, Kollen Boudewijn, Twisk Jos (2006). Impact of Time on Improvement of Outcome After Stroke. Stroke.

[40] Stanton Rosalyn, Ada Louise, Dean Catherine M., Preston Elisabeth (2016). Effect of information feedback on training standing up following stroke: a pilot feasibility study. Topics in Stroke Rehabilitation.

[41] Bower Kelly J, Clark Ross A, McGinley Jennifer L, Martin Clarissa L, Miller Kimberly J (2014). Clinical feasibility of the Nintendo Wii™ for balance training post-stroke: a phase II randomized controlled trial in an inpatient setting. Clinical Rehabilitation.

[42] Lannin NA, Ada L, Levy T, English C, Ratcliffe J, Sindhusake D, et al. Intensive therapy after botulinum toxin in adults with spasticity after stroke versus botulinum toxin alone or therapy alone: a pilot, feasibility randomized trial. Pilot Feasibility Stud. 2018;4:82. 10.1186/s40814-018-0276-6 PMC5963180 29796293

[43] Stinear Cathy M., Byblow Winston D., Ackerley Suzanne J., Smith Marie-Claire, Borges Victor M., Barber P. Alan (2017). Proportional Motor Recovery After Stroke. Stroke.

